# Hybrid flower pollination algorithm strategies for t-way test suite generation

**DOI:** 10.1371/journal.pone.0195187

**Published:** 2018-05-02

**Authors:** Abdullah B. Nasser, Kamal Z. Zamli, AbdulRahman A. Alsewari, Bestoun S. Ahmed

**Affiliations:** 1 Faculty of Computer Systems and Software Engineering, Universiti Malaysia Pahang, Kuantan, Pahang, Malaysia; 2 Faculty of Electrical Engineering, Department of Computer Science, Czech Technical University, Praha 2, Czech Republic; Guangxi University for Nationalities, CHINA

## Abstract

The application of meta-heuristic algorithms for t-way testing has recently become prevalent. Consequently, many useful meta-heuristic algorithms have been developed on the basis of the implementation of t-way strategies (where t indicates the interaction strength). Mixed results have been reported in the literature to highlight the fact that no single strategy appears to be superior compared with other configurations. The hybridization of two or more algorithms can enhance the overall search capabilities, that is, by compensating the limitation of one algorithm with the strength of others. Thus, hybrid variants of the flower pollination algorithm (FPA) are proposed in the current work. Four hybrid variants of FPA are considered by combining FPA with other algorithmic components. The experimental results demonstrate that FPA hybrids overcome the problems of slow convergence in the original FPA and offers statistically superior performance compared with existing t-way strategies in terms of test suite size.

## 1. Introduction

Many aspects of software engineering (e.g., requirements, management, testing, and refactoring) deal with optimization problems. In summary, optimization problems involve exploiting limited resources to find optimal solutions from a potentially large number of alternative solutions. Meta-heuristic-based algorithms excel in this arena. Many meta-heuristic algorithms have been developed in prior studies, including that of tabu search (TS) [1], simulated annealing (SA) [2], genetic algorithm (GA) [3], ant colony algorithm (ACA) [4], particle swarm optimization (PSO) [5], differential evolution (DE) [6], harmony search (HS) [7], flower pollination algorithm (FPA) [8], sine cosine algorithm (SCA) [9], bee algorithm (BA) [10], cuckoo search (CS) [11], and firefly algorithm (FA) [12].

In the field of t-way testing, meta-heuristic algorithms have been used to sample an optimized set of test suites from large combinatorial values on the basis of a specified interaction strength (t). However, the main issue involves the identification of optimal test cases from an exhaustive test suite. The searching operation for the optimal set of test cases is a non-deterministic polynomial-time hard (NP-hard) problem in which additional software components can exponentially increase computational time and problem complexity. To address this issue, many studies have adopted meta-heuristic algorithms on the basis of their implementation, including TS [13], SA [13], GA [13,14], CA [14], PSO [15], HS [16], and CS [17]). However, although useful, these strategies have limitations.

Strategies based on TS and SA often produce optimal results for a small set of test configurations, but they are prone to being limited to the local minimum solution [16]. Although useful, strategies based on GA, ACA, PSO, and HS often require frequent interactions with the environment during computation. For instance, GA exploits crossover and mutation operators with historical information to explore regions with relatively better solutions. ACA requires the indirect communication of a colony via pheromone trails, while PSO similarly interacts with individual particles through velocity updates in a given swarm until the solution is reached. HSS requires the use of probabilistic values from the pitch adjustment rate (PAR) and the harmony memory considering rate (HMCR) to select the solution from the harmony memory (HM) or regenerate a new random solution. Nonetheless, PSO and HSS can address the limitations of GA and ACA in terms of supporting high-interaction strength (i.e., t ≥ 6).

Although useful, the capability of existing t-way strategies remains limited given that no single strategy appears to be superior compared with other configurations [18]. To address the shortcomings, the search for a new t-way strategy that considers a new breed of search techniques is justified. Two algorithms can be hybridized by compensating the limitation of one algorithm with the strength of others. Conferences, workshops, and review papers on hybridization have shown that hybridization topics have since become extremely popular [19]. In fact, many studies have reported that hybrids of optimization-based algorithms often perform better than their original algorithmic counterparts [20].

In accordance with the aforementioned prospects, this paper presents hybrid variants of strategies for t-way test suite generation on the basis of a new meta-heuristic called the FPA [8]. The adoption of FPA is justified by the advocacy of many recent studies of its superiority over GA, PSO, and HS [21,22]. Additionally, FPA also offers the following advantages:

- FPA offers a simple flower analogy with lightweight computation based on only one control parameter (i.e., switch condition, *p*) unlike GA, HS, and PSO.
- FPA offers a balanced intensification and diversification of solutions through the adoption of lévy flight (i.e., random walks that are interspersed by long jumps) and switch condition *pa*, which can be used to change between global search and intensive local search.

Although proven efficient, FPA is prone to being restrained to the local optima due to the weakness of having to use a diverse population [23–26], especially for multimodal optimization problems. To overcome this weakness, many FPA hybridizations have been proposed. This paper investigates four FPA hybridizations for the t-way test suite generation. Our hybridization approach is unique given that we adopt peer efficient components (i.e., elitism feature, mutation operator, and local search) as our main hybridization constructs.

The rest of this paper is structured as follows. Section 2 presents an overview of the t-way testing and its theoretical background. Section 3 provides a review of existing strategies. Section 4 presents a detailed review of FPAs and their applications. Section 5 explores four FPA hybridization variants for the t-way test generation. Section 6 discussed the experiment and results. Section 7 discusses the threats to validity. Section 8 concludes the present research with recommendations for future work.

## 2. Background

### 2.1 T-way test suite generation problem

The sampling technique called t-way testing generates test cases that focuses on the behavior of interacting system components. To illustrate the concept of t-way testing in test suite reduction, we consider a hypothetical online payment service as an example. Online payment allows the electronic exchange of money, in which customers are instructed to fill out an online payment form and submit the required information to the merchant’s website. The form consists of six parameters (i.e., payment method, name on card, card number, expiration date (with the two inputs of MM and YY), and card CVV). Five payment methods exist (i.e., “Visa Card,” “Master Card,” “American Express,” “Discover,” and “PayPal”).

**Fig 1:**
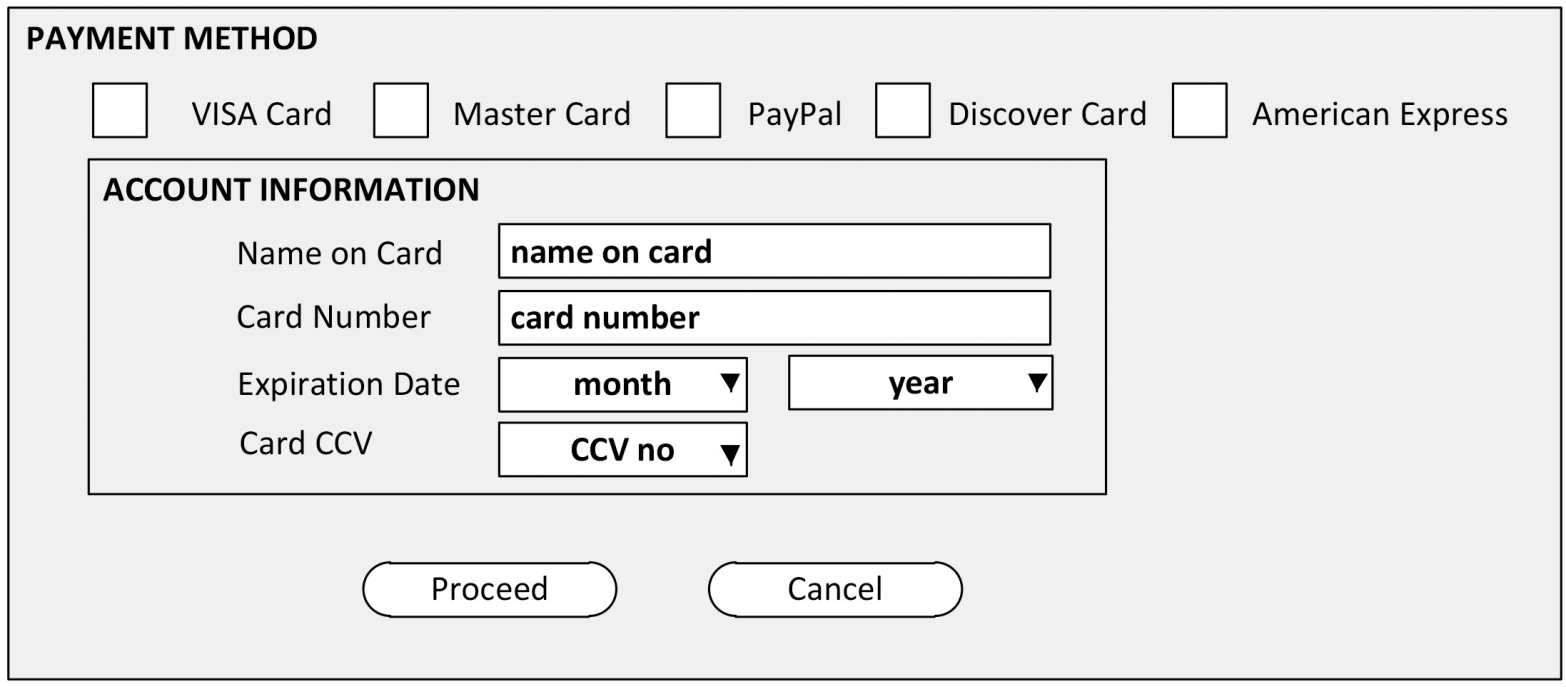
Example of hypothetical online payment.

As shown in Fig 1, “Name-On-Card” and “Card-Number” use one string value each; “Expiration-Date” is considered as two inputs (i.e., MM takes a value from 1 to 12, and YY takes a value from 16 to 31); and Card CVV uses one input value.

A total of 900 test cases are required to fully test this system. In this case, the two-way test suite requires only 180 test cases, thereby saving 80% in time and effort. As the interaction increases, the number of t-way test suite increases toward the exhaustive set. In general, every t-combination of input values (where t indicates the interaction strength) is covered by the test case at least once [16,27]. Studies on NASA application show that 67% of failures can be detected if a single parameter value is at least tested (interaction strength t = 1), 93% of failures can be detected if all pairs of parameter combinations are tested (interaction strength t = 2), and 98% of failures can be detected if all 3-tuple interactions are tested (interaction strength t = 3). In addition, the fault detection rate for the other applications can reach 100% if the interaction strength (t) is between 4 and 6 [28–32].

### 2.2 Theoretical background

The test suite (T) is an *n*×*m* array of *n* rows of generated test cases wherein each test case is a combination of *m* input values. A t-way test suite (T1) covers every valid pair of input parameters, wherein one test case can cover many pairs of input values. The t-way problem involves finding the effective test suite (T1) from T that has the smallest number of rows.

**Definition 1**: (t-way Test Suite): Given a set of N parameters, *P*_*1*_, *P*_*2*_,…*P*_*n*_, each of which has *v*_*i*_ possible values [*v*_*1*_, *v*_*2*_,…*v*_*m*_], the t-way test suite of strength *t* is an *N×n* array, such that each column contains only elements from *v*_*i*_ and every *N×t* sub-array contains all combinations of size *t* at least once.

Covering array (CA) is a mathematical object that is often adopted to describe the generated t-way test suite [33,34]. In general, any system under test (SUT) comprises several components called parameters that interact with each other with their associated values. In this paper, v, p, and t denote number of parameters, associated levels, and interaction strength, respectively. When the number of values (*v*) is equal for all parameters (*p*), the CA is represented as the uniform CA(*N*, *t*, *v*^*p*^). For example, CA(6; 2, 2^4^) consists of six rows of test cases that are generated from four columns of parameters with two values each. When the number of parameters are not equal (i.e., each parameter has a different number of values), the CA representation takes the mixed CA notation of MCA(*N*, *t*, *v*_*1*_^*p1*^ *v*_*2*_^*p2*^ *v*_*3*_^*p3*^…‥*v*_*j*_^*pj*^). As an additional example, MCA (12, 3, 2^3^ 3^1^) represents a test suite that consists of arrays with 12 rows and 4 columns of parameters, in which three parameters have 2 values and one parameter have 3 values.

## 3. Related work

In general, t-way strategies can be classified into two main algebraic and computational approaches [16,35]. In algebraic approaches, test sets are constructed without enumerating any combinations because they are based on lightweight computations. Strategies of this approach, including orthogonal Latin squares (OLS), CA, MCA, and test configuration (TConfig), are often restricted to small configurations [15,36]. Computational approaches use greedy algorithms to construct test cases to cover as many uncovered combinations as possible. These approaches generate the incremental test suite either using the one-parameter-at-a-time or one-test-at-a-time approach.

One-parameter-at-a-time strategies start by building a complete test suite for the first two parameters or the smallest number of interaction components, then extends horizontally by adding one parameter per iteration, and sometimes extends vertically until all parameters are covered. The most well-known strategy of this approach is the in-parameter-order (IPO) strategy [37]. On the basis of the IPO strategy, many improvements, such as IPOG [38], IPOG-D [35], IPOF, and IPAD2 [39], have been proposed. One-test-at-a-time strategies build a single complete test case per iteration until all interaction elements are covered. The automatic efficient test generator (AETG) proposed by Cohen et al. [40] is considered the first attempt to adopt this approach. Subsequently, many tools and strategies have been proposed by researchers, such as Jenny [41], TConfig [42], and WHITCH [43].

Many researchers have recently adopted meta-heuristic search algorithms, such as HC, TS, SA, GA, ACA, HS, and CS, on the basis of t-way test suite generation. HC is perhaps the most basic search algorithm for successfully generating a two-way test suite, but is sensitive to the initial search position and hence susceptible to being restrained to the local optima. TS has also been used successfully for two-way test suite generation. SA, an improvement of HC, allows movement to poor solution, with some probability, even though the best solution has been reached (i.e., to avoid being restrained to the local minimum). SA has been implemented for three-way interaction test suite generation unlike HC and TS. Meanwhile, GA [13,14,44] and ACA are early studies on adopting population-based algorithms to generate t-way test suites. GA starts by finding solutions from many positions unlike HC, TS, and SA. Therefore, the chances of reaching optimum solutions are high. The main advantage of GA over HC, TS, and SA is that it is not usually restrained in the local optima. Moreover, GA provides some control in the selection processes, such as genetic diversity and selective pressure, to ensure an adequately diverse population.

PSO has been adopted in the particle swarm-based test generator (PSTG) strategy [15] and the variable strength t-way test suites generation (VS-PSTG) strategy [45]. PSO is a population-based strategy that mimics the behavior of birds and fishes in a swarm when searching for food. Unlike GA and ACA, the PSO-based strategy can support high-interaction strengths that can reach t = 6, but its computation time is relatively longer in practical usage [46]. HS has been adopted in the harmony search-based strategy (HSS) for implementing and generating t-way test suites. Using HSS, the test data generation process mimics the improvisation process of a skilled musician [16]. Furthermore, HSS uses a sort of elitism and/or the selection used in GA to efficiently explore the search spaces [47] and a probabilistic-gradient to select the current solution neighbor, while mathematical equations are used to move toward finding the relatively better solutions [48].

CS is a population-based algorithm inspired by the brood parasitic behavior of birds, such as Ani and Guira cuckoos [42]. CS provides an optimal balance between local intensification and global diversification by intensifying the solution search process in the neighborhood of incumbent solutions and efficiently explores the entire search space using lévy flights [43]. Similar to HS and GA, CS adopts elitism mechanisms to ensure that only solutions with high fitness can move toward the next generation.

With regard to the hybridization of meta-heuristics and its application for t-way strategies, several existing studies can be highlighted. Zamli et al. [49] proposed the hybrid meta-heuristic variant called high-level hyper-heuristic (HHH), which explores the concept of hyper-heuristics wherein a master heuristic can choose from more than one (slave) heuristics. In their work, Tabu search (TS) serves as the master algorithm (i.e., high level) that controls the following four other low-level algorithms (LLH): teaching—learning-based optimization, PSO, CS, and global neighborhood algorithm. During runtime, HHH adopts three operators (i.e., diversification, intensification, and improvement) to decide on the best low-level algorithm for any particular running instance. Although useful in enhancing the diversification and intensification of the entire search process, the hybridization approach based on the HHH is bulky and computationally heavy. Furthermore, each LLH requires extensive tuning, without which poor performance may ensue.

## 4. Flower pollination algorithm

**Fig 2:**
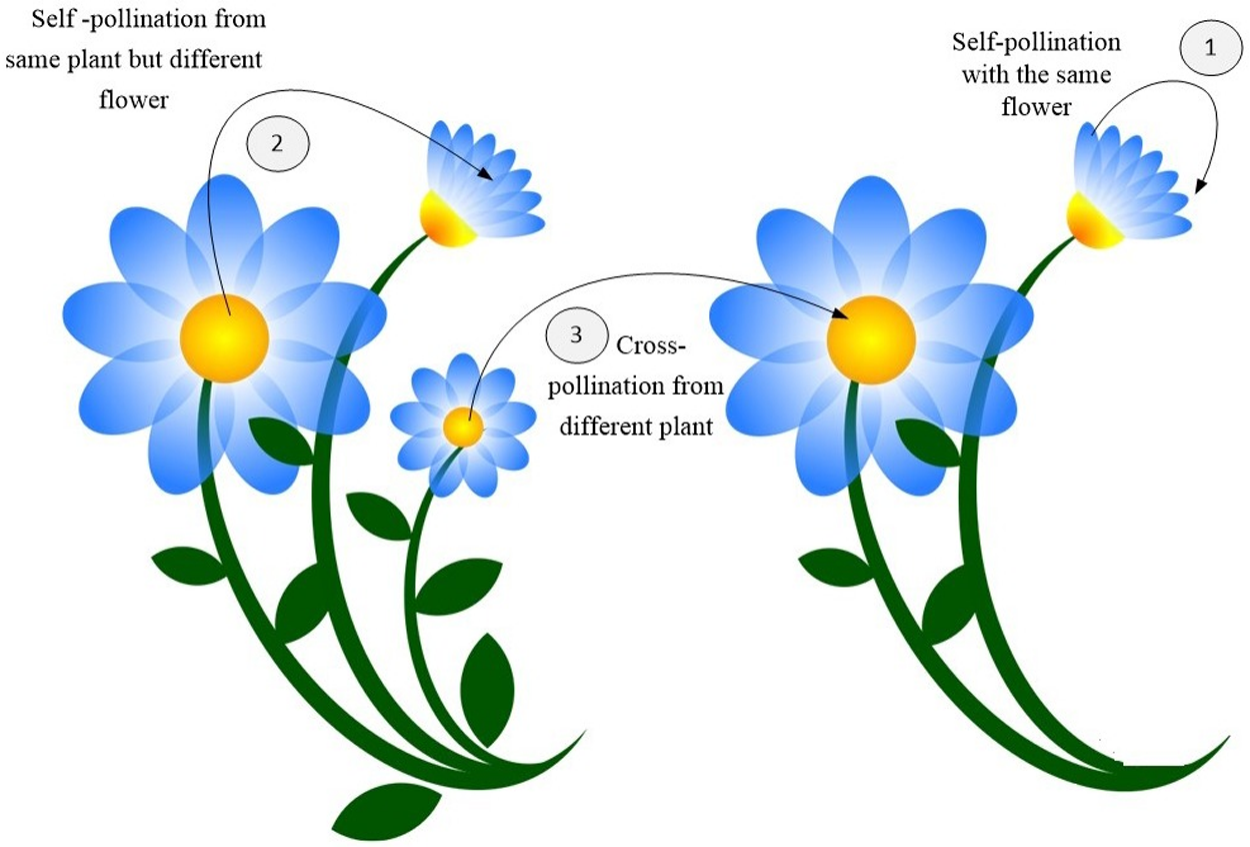
Flower pollination methods. (1) Self-pollination with the same flower, (2) Self-pollination from same plant but different flower, and (3) Cross-pollination from different plant.

FPA is one of the latest meta-heuristic algorithms inspired by the pollination behavior of flowering plants. Pollination involves transferring pollen grains from the male part of the flower to ovules borne in the female part via pollinators, such as birds, butterflies, bees, and bats. According to the mechanisms of pollen transfer, pollination can take two types: biotic and abiotic. Biotic pollination refers to the transfer pollen via pollinators (i.e., insects or other animals). By contrast, abiotic pollination does not require any pollinators to transfer pollen (i.e., uses non-animal vectors, such wind and water). Furthermore, pollination can be accomplished by self-pollination or cross-pollination. Self-pollination occurs when the pollen is transferred from the male to the female parts of the same flower or to another flower of the same plant. Cross-pollination refers to the transfer of pollen from the flower of one plant to the flower of another plant (Fig 2) [50].

Some flowers facilitate or even restrict specific pollinators, and such flowers often use many methods, such as colors, scents, petals, and nectars, to attract pollinators. The tendency to specialize in this manner is referred as “flower constancy,” a term to define the preference of many pollinators to visit only certain species of flowers and ignore alternative flowers. The main advantage of flower constancy is maximized pollen transfer, which in turn increases the reproduction of the corresponding flower [51].

### 4.1 Basic form of flower pollination algorithm

Based on the characteristics of flower pollination (i.e., pollination process, flower constancy, and pollinator behavior), FPA can be represented mathematically by two key steps: global and local pollination. The global pollination step in FPA is represented by the transfer of flower pollens by pollinators (such as insects) over a long distance, and this approach guarantees that the fittest pollens with high quality are carried over to the next generation.

$${x_{i}}^{\left(t+1\right)}\;=\;{x_{i}}^{\left(t\right)}\;+\;\gamma Lévy\;(\lambda )(X_{t}\;-\;gbest)$$ (1)

where x_i_^(t)^ is the ith pollen or solution at iteration t, *gbest* is the current best solution, γ>0 is the step size, and Lévy (λ) is lévy flight. Lévy flight, which is used to efficiently mimic the characteristic of long-distance movement of insects, is essentially a random walk interspersed by long jumps distributed to different regions according to a power law.

Local pollination and flower constancy (achieved by abiotic pollination) is formulated by the following equation:

$${x_{i}}^{\left(t+1\right)}\;=\;{x_{i}}^{\left(t\right)}\;+\;\epsilon ({x_{j}}^{\left(t\right)}\;-\;{x_{k}}^{\left(t\right)})$$ (2)

where x_j_^(t)^ and x_k_^(t)^ are pollens selected randomly from different flowers, while ϵ is a random number that follows the uniform distribution in [0,1]. Eq 1 mimics the characteristic of self-pollination and abiotic pollination based on flower constancy.

**Fig 3:**
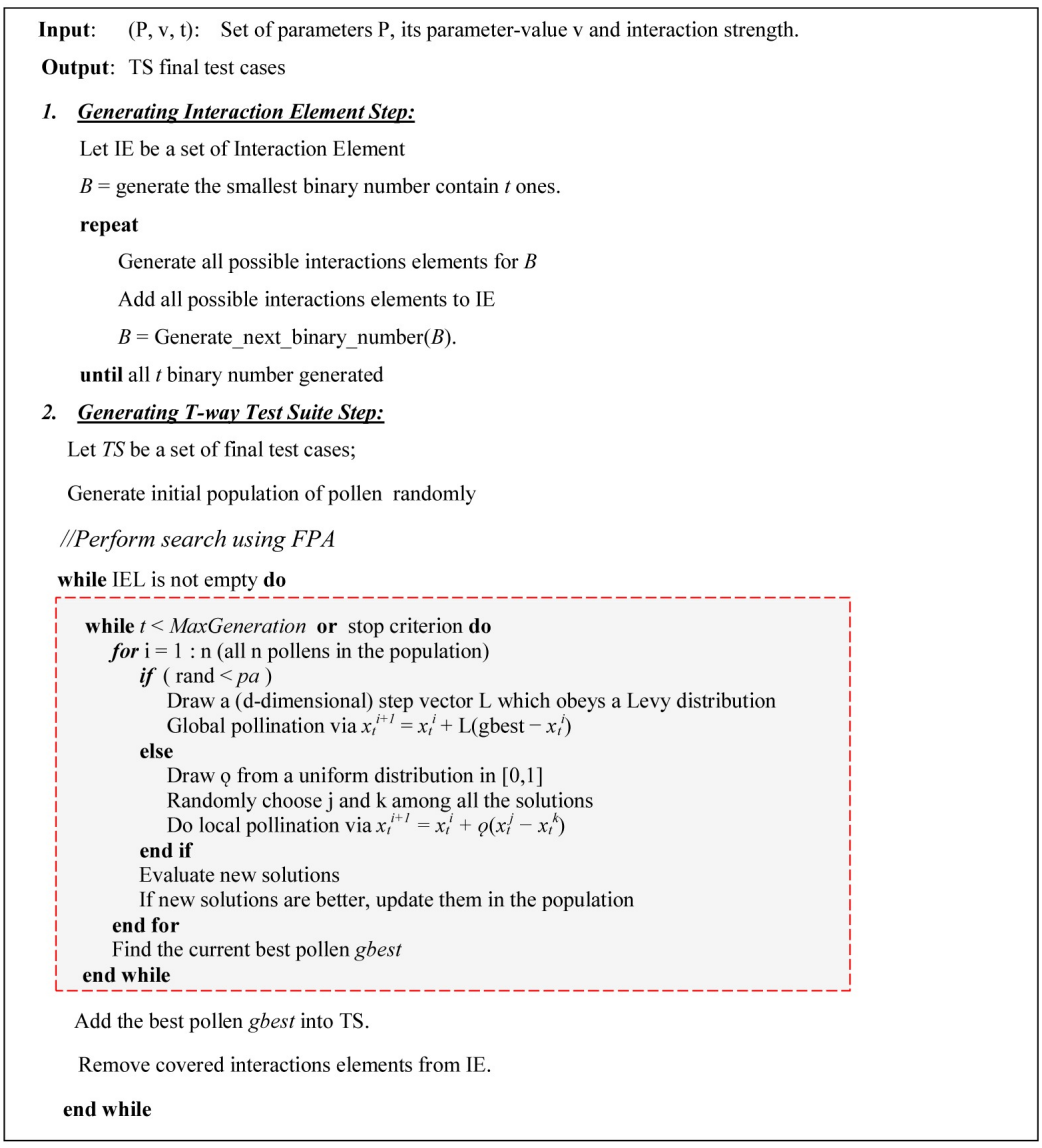
FPA strategy for t-way test suite generation.

In general, FPA begins by randomly initializing the flower pollen population or solutions. For each algorithmic generation, a new solution is generated using either global pollination or local pollination, which is controlled by a switch probability *pa* ϵ [0, 1]. The summary of FPA is illustrated in the shaded box in Fig 3.

### 4.2 Hybrid flower pollination algorithm

Many FPA hybridization variants have also been proposed in the literature, including the chaotic HS for solving Sudoku puzzles [52], FPA with GA for solving constrained optimization problems [53], FPA with PSO (FPAPSO) for solving constrained global optimization problems [54], FPA with TS for solving unconstrained optimization problems [55], FPA with DE (DE-FPA) to overcome the drawbacks of slow convergence to global optima [56], FPA with clonal selection algorithm [57], and FPA with artificial bees and biogeography optimization algorithm for satellite image classification [58]. Recently, DE-FPA has also been integrated with the time-varying fuzzy selection mechanism to find the optimal dispatch of wind—thermal dynamic multi-objective problems [25]. In other words, FPA with randomized location and crossover has been proposed to enhance population diversity [24]. Wang and Zhou [59] improved the convergence speed of FPA to adopt the dimension-by-dimension evaluation and local neighborhood operator, while Zhou et al. [26] adopted the elite opposition technique to select the optimal solution. Wang et al. [23] adopted three new operators for the FPA, namely, the discard pollen, elite-based mutation, and crossover operators, while Zhou and Wang [60] adopted the dynamic switching probability strategy and proposed the FPAPSO for the optimal path planning of unmanned undersea vehicles.

Although useful, most of the existing FPA hybridizations highlighted take the maximalist approach, that is, embed the complete meta-heuristic algorithm with FPA, thereby altering its original structure and/or adding new control parameters. In the present work, we adopt a minimalist approach to maintain the original FPA structure in our hybridization.

## 5. Flower pollination algorithm based strategy for t-way test suite generation

This section describes the design and implementation of the proposed strategy based on the original FPA, called the FPA strategy. The FPA strategy uses the original FPA to generate an optimized test suite by searching test cases that cover maximum numbers of t-combinations. In the FPA strategy, each test case can be treated as a pollen or feasible solution and the interaction element as the search space. At the start, FPA generates the list of all interaction elements stored in the population of pollens. Then, during the evaluation loop, the population of pollens is repeatedly subjected to the FPA’s search cycle to construct an optimized test case for the test suite.

To address the problem of t-way test suite generation, FPA adopts two major steps: (A) generating the interaction element and (B) generating the t-way test suite (Fig 3). These two steps are explained in detail in the next sections.

### A. Generating interaction element

To generate the interaction elements for a set of parameter (P) and their values (*v*), all possible binary combinations of P-digit are generated, and then the binary combinations that contain 1’s equal to the interaction strengths, *t*, are selected. Here, each parameter in the system is represented by a digit (0 or 1), where 0 indicates the exclusion of parameter and 1 indicates the inclusion of parameter. Therefore, binary combination 1100 refers to the P_1_P_2_ parameter combination and binary combination 1011 refers to the P_1_ P_3_ P_4_ parameter combination. As illustrated, considering a system with four parameters (P1, P2, P3, and P4), variable strength configuration VCA (N; 2, 2^3^ 3^1^, [CA (3, 2^3^)]) indicates four parameters with t = 2 for the main configuration with three parameters, with each having two values (0 and 1) and one parameter having three values (0, 1, and 2), and t = 3 for three parameters with two values as the sub configuration. For the main configuration *t* = 2, the binary combinations that only contain two ones (i.e., 1100, 1010, 1001, 0110, and 0101) are generated and added to the binary combinations set. For the sub-configuration t = 3, the binary combinations that contain three ones are also added to the binary combinations set.

Based on the generated binary combinations, FPA begins to generate the interaction elements list. For our running example, P1, P2, and P3 have two values (i.e., 0 and 1), and P4 has three values (0, 1, and 2). For each binary combination, all possible combinations of the corresponding parameter values are added to the IE. For instance, binary combination 1100 (refers to P1, P2) has 2×2 possible interaction elements (i.e., 0:0, 0:1, 1:0, and 1:1), while 1001 (refers to P1, P4) has 2×3 possible interaction elements (i.e., 0:0, 0:1,1:0, 1:1, 2:0, and 2:1).

### B. Generating t-way test suite

The t-way test suite is a set of test cases that cover the interaction elements. The FPA attempts to generate an optimal test suite that covers all interaction elements at least once. The FPA begins by initializing population size *pollen size*, probability *pa*, and stopping criteria (i.e., maximum iteration for improvement). Then, the FPA generates and evaluates the *pollen size* of the pollen population randomly. Here, the fitness value of each pollen is the number of interaction elements that are covered by the pollen. Subsequently, in each generation of the algorithm, the pollen population is subjected to repeated cycles of the FPA search process. In general, one of the two core operations is performed on the population of pollens. The first core part of the algorithm generates a new pollen, *x*^*new*^ = (*x*_1_^*new*^, *x*_2_^*new*^, …, *x*_*n−*1_^*new*^, *x*_*n*_^*new*^), using global pollination (i.e., lévy flight as expressed in Eq 1). Based on the new pollen’s weight, the new pollen is determined whether it is the current pollen. The second core part of the algorithm is the local pollination process. In the local pollination, two test cases are randomly selected from different flowers to generate a new test case as demonstrated by Eq 2.

The search process is repeated until the maximum number of improvements is achieved (i.e., in this case, the best test case covers the most interaction elements) or the candidate solution weight is equal to the maximum weight that can be covered. In both cases, the FPA adds the best pollen into the final test suite, and then the covered interactions elements are removed from the interaction list. Subsequently, the interaction elements list is checked. Once all interaction elements are covered (i.e., the interaction list is empty), the iteration stops. Otherwise, the search process is repeated.

### 5.1 Parameter tuning of the FPA

**Fig 4:**
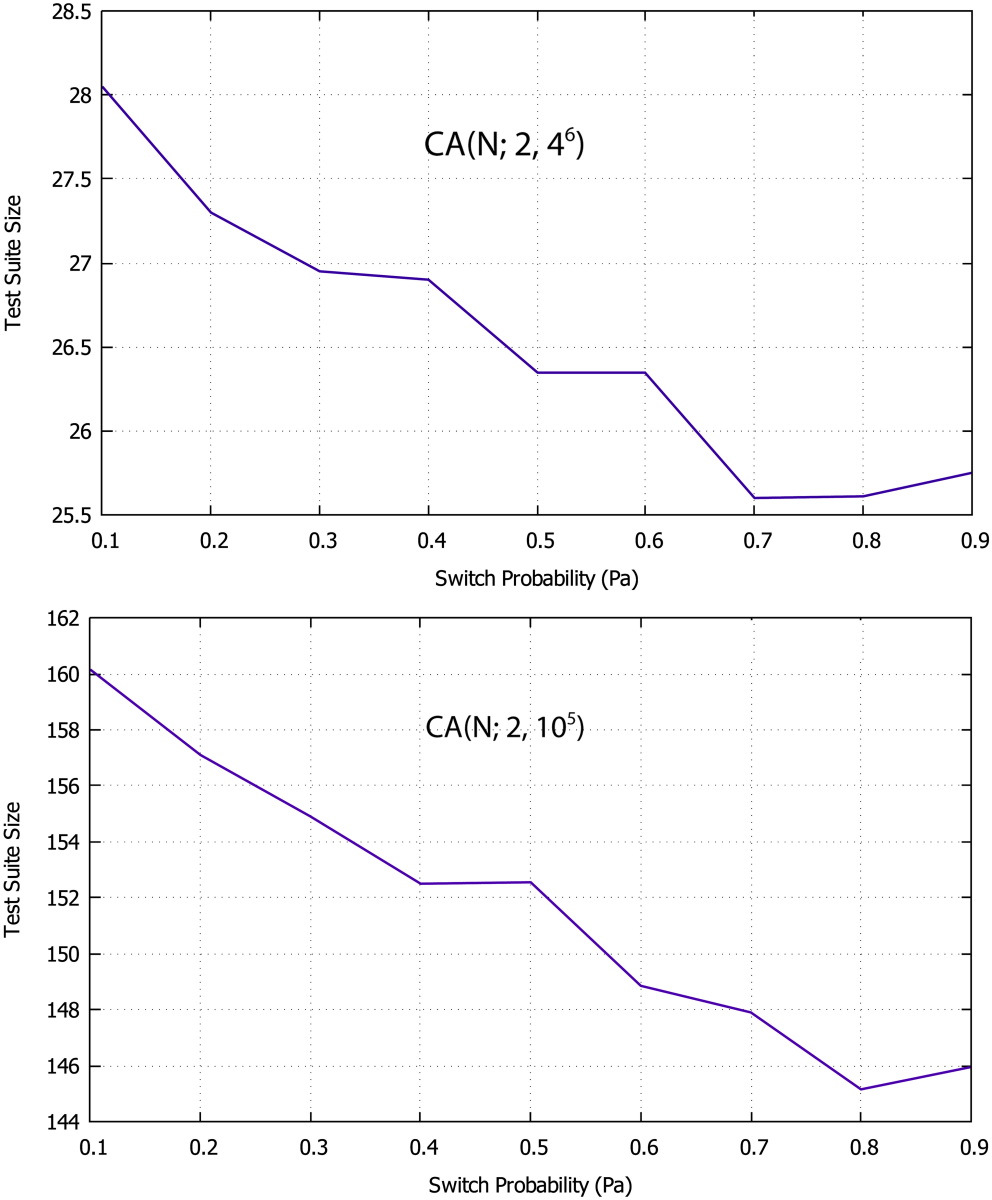
Graphical representation of averages test suite for CA(N; 2, 4^6^) and CA(N; 2, 10^5^) with pollen size = 10, and iteration = 30.

**Fig 5:**
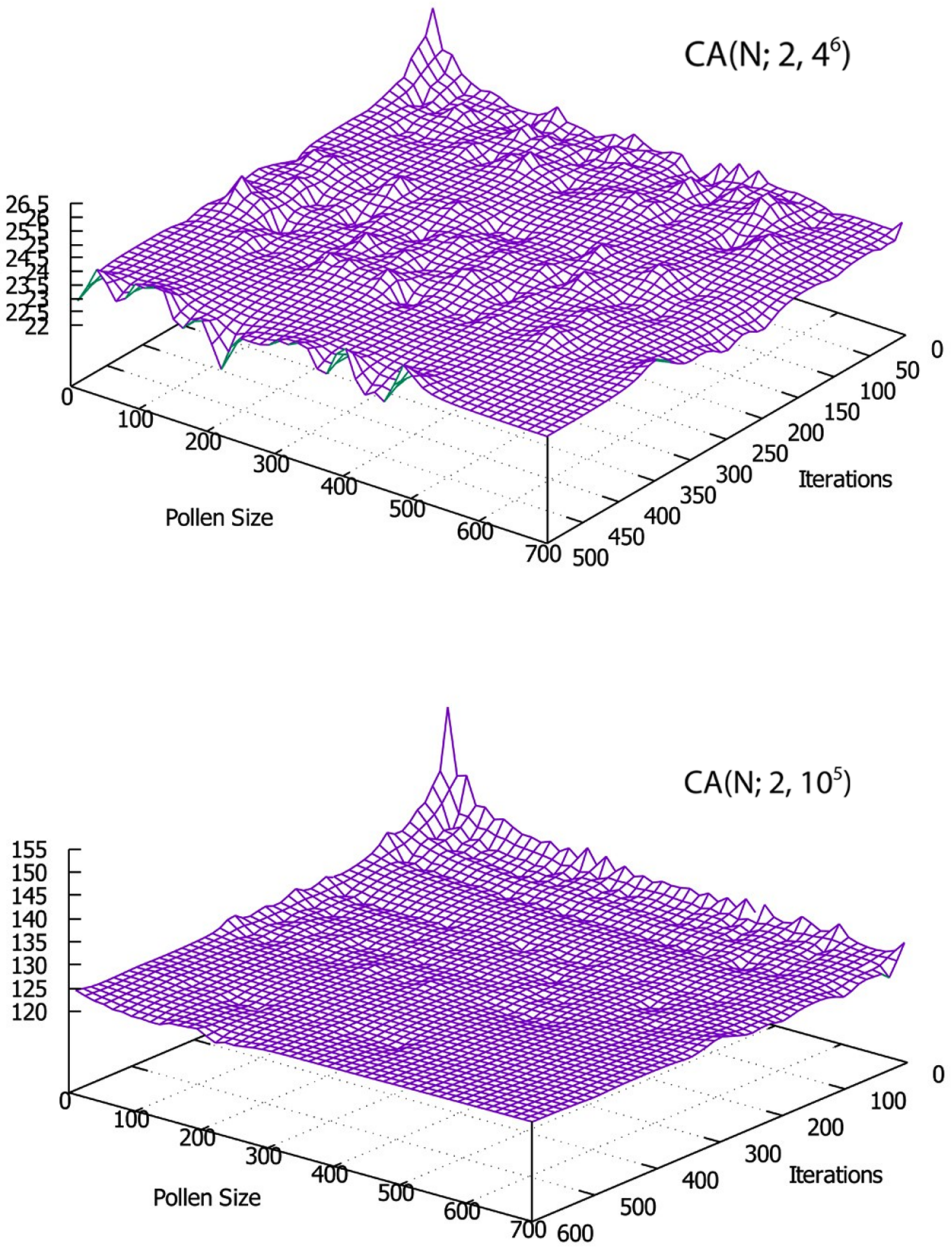
Graphical representation of averages test suite for CA(N; 2, 4^6^) and CA(N; 2, 10^5^) with switch probability *pa* = 0.7.

The behavior of the FPA is largely determined by population size *pollen* s*ize*, switch probability *Pa*, and iteration number *n*. Therefore, these parameters may require tuning. To this end, two well-known CAs, CA (N; 2, 4^6^) and CA (N; 2, 10^5^), are used [15,16]. For systematic tuning, we fix the values of two parameters and try different values for the third parameter. For example, the value of pollen sizes and iterations are fixed (i.e., pollen size = 10 and iteration = 30) and various values of *Pa* (i.e., 0.1, 0.2, 0.3, … 0.6) are tested as shown in Table 1 and Fig 4. Then, the reverse process is performed for each parameter as shown in Tables 2 and 3, and Fig 5 respectively. Here, the FPA is executed 20 times for every parameter value, and the average value is taken from the results.

**Table 1:** Averages test suite for CA(N; 2, 4^6^) and CA(N; 2, 10^5^).

Covering Array	Switch Probability (*pa*)								
	0.1	0.2	0.3	0.4	0.5	0.6	0.7	0.8	0.9
CA (N; 2, 4^6^)	28.05	28.05	26.95	26.9	26.35	26.35	25.6	25.61	25.75
CA (N; 2, 10^5^)	160.15	157.1	154.9	152.5	152.55	148.85	147.9	145.15	145.95

**Table 2:** Averages test suite for CA(N; 2, 4^6^).

Pollen Size	Iteration										
	5	10	20	30	40	50	100	200	300	500	700
10	35.65	32.15	32.95	30.30	29.95	29.15	28.55	27.65	26.40	25.65	25.25
20	32.20	29.65	28.80	28.45	27.55	27.40	26.95	25.10	24.95	24.80	24.50
30	30.95	28.65	28.00	26.80	26.75	25.50	25.35	24.65	24.50	24.20	24.00
50	29.05	26.65	27.20	25.95	25.95	25.15	25.55	24.10	24.00	24.00	24.00
100	27.15	26.10	25.65	25.25	24.65	24.70	24.55	23.85	24.10	23.90	23.50
200	26.25	25.15	24.80	24.65	24.50	24.35	24.00	23.40	23.50	23.90	23.75
300	25.90	24.90	24.55	24.05	24.45	24.15	23.95	23.80	23.70	23.45	24.00
500	24.80	24.50	24.15	24.05	23.70	23.80	24.10	23.35	23.55	23.80	23.65

**Table 3:** Averages test suite for CA (N; 2, 10^5^).

Pollen Size	Iteration										
	5	10	20	30	40	50	100	200	300	500	700
10	146.4	138.7	134.9	133.6	131.3	128.2	127.4	127.2	126.2	126.3	125.3
20	138.3	132.6	131.2	128.9	128.4	126.0	125.5	124.1	124.3	124.6	124.6
30	135.8	130.7	128.4	127.9	125.9	125.0	124.3	124.2	124.2	123.4	123.9
50	133.0	129.1	126.7	125.5	125.5	123.5	123.7	122.8	122.8	123.0	123.6
100	129.3	126.5	125.0	123.9	123.7	123.3	123.4	123.2	123.4	123.3	123.5
200	126.8	125.0	123.8	123.7	123.8	122.1	122.8	123.3	122.6	122.6	122.6
300	125.4	124.5	123.8	123.7	123.6	123.2	122.8	122.7	123.3	123.6	122.5
500	124.4	124.1	123.3	123.1	123.0	123.2	123.0	123.1	122.6	122.8	122.8

Referring to the results shown in Tables 2 and 3, it can be observed that using large value of pollen size may lead to better results, and conversely using too small value may lead to poor results. By increasing the number of pollens up to 30, the performance of the FPA strategy is improved. However, a high pollen value (i.e., equal to 500) does not necessarily yield better results. The best results are obtained when the number of pollen is between 50 and 100. Otherwise, the iteration value increases and the result improves. The best result is obtained when the iteration value varies from 300 to 500. In terms of switch probability (*pa)*, the results show that using a higher *pa* can lead to better results. However, when *pa* is between 0.8 and 0.9, the proposed strategy obtains good results.

Therefore, the FPA generally obtains the optimal test suite when pollen size is between 50 and 100, the repetition is between 300 and 500, and *pa* is between 0.8 and 0.9.

### 5.2 Hybrid FPA-based strategies for t-way test suite generation

The original FPA-based method for test suite generation has two core components: global pollination via lévy flight and local pollination. The FPA performance may be enhanced by adding one or more components from other efficient algorithms to the FPA. Here, we present three components that will be injected into the FPA. These three components have been carefully selected to improve the FPA’s intensification and diversification.

- *Elitism Feature*: Elitism is a simple way of improving the efficiency of randomization, that is, a good candidate solution is retained (and the poor ones are randomly replaced from the population) to be carried over to the next iteration.
- *Mutation operator*: Mutation maintains the diversity solution of the population from one generation to the next one (i.e., as one or more solution values are changed). In our work, we adopt the bit string mutation.
- *Local Search*: This is a simple and highly effective technique for finding a local optimum solution. Local search only moves from current states to neighboring states if they improve the current solution.

**Fig 6:**
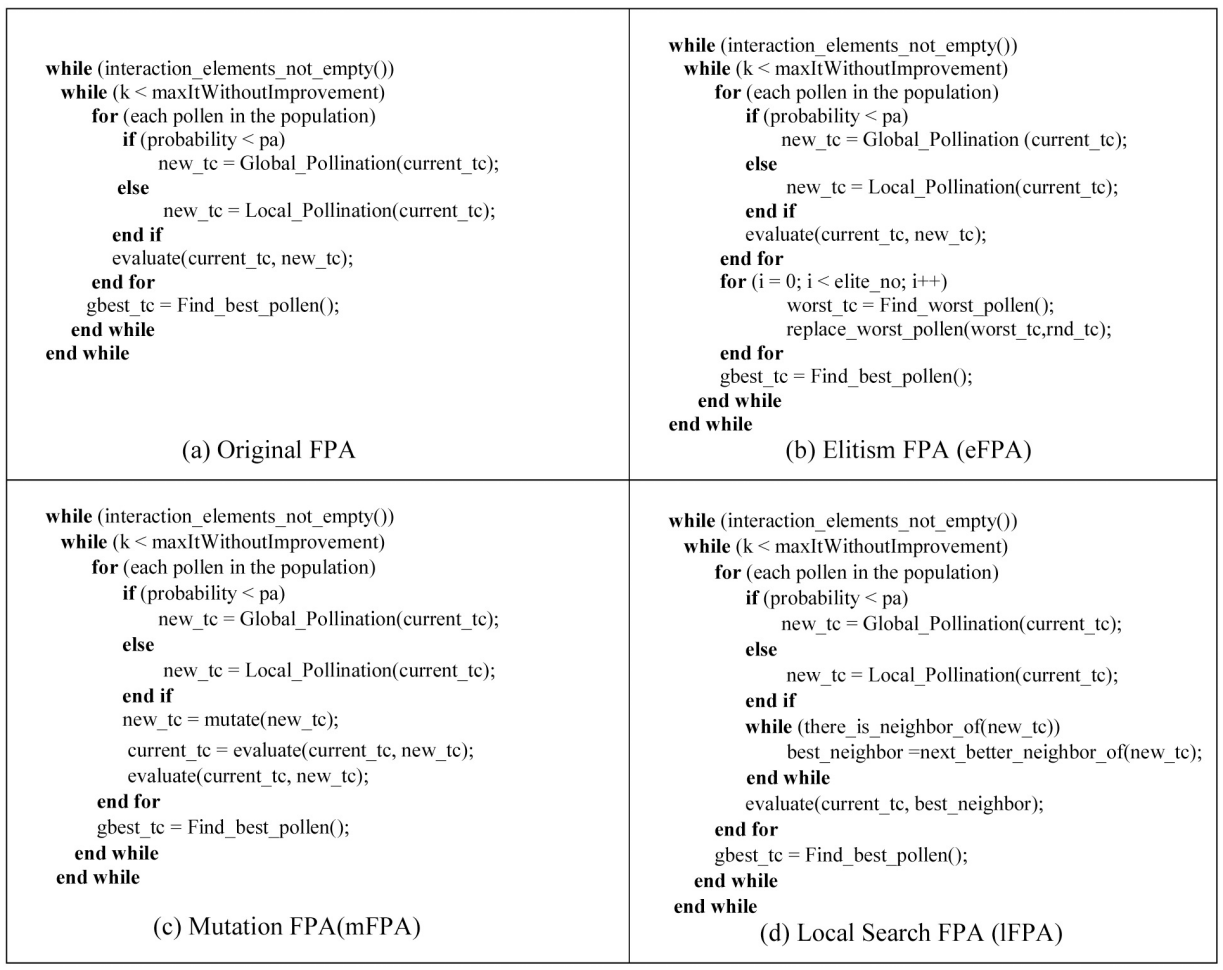
Hybridization variants of FPA.

The hybridization of FPA with other components can occur in every component of the standard FPA. In this paper, we propose four variants of FPA: original FPA, hybrid elitism FPA (eFPA), hybrid mutation FPA (mFPA), and hybrid local search FPA (lFPA). The hybrid eFPA variant uses the elitism technique to retain the elite population and replace the poor population by a new pollen randomly. The hybrid mFPA variant uses the mutation operator to include diversity in the population of pollens. The hybrid lFPA uses intensive local search to improve local intensification. The complete excerpt pseudo code variants for the original FPA, hybrid eFPA, hybrid mFPA, and hybrid 1FPA are highlighted in Fig 6.

## 6. Experiments and evaluation

Our experiments are based on three related goals. First, we evaluate the efficiency of the proposed strategies to select the best hybrid variant FPA in comparison with the existing work. Second, we benchmark the best hybrid variant against other existing strategies. Finally, we verify our findings using statistical analysis. The results are displayed in tables and graphs. The experiments are performed on Core i7-3770 CPU@ 3.40 GHz, Windows 7 professional machine. We adopted 20 runs for each experiment for statistical significance.

For the parameters setup, we adopted the tuned FPA parameters as discussed in Section 5.1. For the other component parameters, such as mutation rate and elitism probability, we took the recommended values (i.e., mutation rate = 0.03 and elitism probability = 0.25) as published in [61]. For a clear perspective, Table 4 depicts the parameters that are adopted for the meta-heuristic strategies [16,17,49] in our experiments.

**Table 4:** Parameters for meta-heuristic strategies of interests.

Algorithm	Parameter	Values
GA	Iteration	1000
	Population size	25
	Best cloned	1
	Random crossover	0.75
	Tournament selection	0.8
	Max stale period	3
	Mutation rate	0.03
	Escape mutation	0.25
SA	Iteration	1000
	Cooling schedule	0.9998
	Starting temperature	20
ACA	Iteration	1000
	Number of ants	20
	Pheromone control	1.6
	Pheromone persistence	0.5
	Heuristic control	0.2
	Pheromone amount	0.01
	Initial pheromone	0.4
	Max stale period	5
	Elite ants	2
PSO	Iteration	100
	Population size	80
	Inertia weight	0.3
	Acceleration coefficients	1.375
CS	Iteration	100
	Population size	100
	Probability *ep*	0.25
HS	Improvisation	1000
	Harmony memory size	100
	Harmony memory consideration rate	0.7
	Pitch adjustment rate	0.2
HHH	Iteration	100
	Population size	40
	Tabu_max_	4
	Inertia weight	0.3
	Acceleration coefficients (c_1_,c_2_)	1.375
	Probability *p*	0.25

Tables 5 through 9 show the results obtained for the experiments. Each cell indicates the minimum test suite size obtained by the existing strategies. Shaded cells denote the best test size obtained by the corresponding strategy, while cells marked as NA denote the unavailability of results in the literature.

**Table 5:** Assessment of hybrid variants of FPA.

Hybridization	CA(N; 2, 10^5^)			CA(N; 2, 4^6^)			CA(N; 3, 5^6^)		
	Avg	Best	Time(s)	Avg	Best	Time(s)	Avg	Best	Time(s)
FPA	127.15	125	17.004	23.80	22	1.955	44.30	42	11.953
eFPA	124.40	122	26.194	22.85	22	3.327	43.53	42	19.264
mFPA	126.25	124	27.900	23.50	22	3.367	44.25	42	21.968
lFPA	127.10	126	18.237	23.70	22	1.912	44.30	42	11.790

**Table 6:** Comparison with existing strategies for different CA and MCA configurations.

No.	Configuration	Computational-based Strategies					Meta-heuristic-based Strategies							
		mAETG	AETG	IPOG	Jenny	TVG	SA	ACA	GA	PSO	HSS	HHH	CS	eFPA
S1	CA(N; 2, 3^4^)	9	9	9	10	11	9	9	9	9	9	9	9	9
S2	CA(N; 2, 3^13^)	17	15	20	20	19	16	17	17	17	18	17	20	17
S3	CA(N; 2, 10^10^)	NA	NA	176	157	208	NA	159	157	NA	155	NA	NA	150
S4	CA(N; 2, 15^10^)	NA	NA	373	336	473	NA	NA	NA	NA	342	NA	NA	333
S5	CA(N; 2, 5^10^)	NA	NA	50	45	51	NA	NA	NA	45	43	42	NA	42
S6	CA(N; 3, 3^6^)	38	47	53	51	49	33	33	33	42	39	33	43	38
S7	CA(N; 3, 4^6^)	77	105	64	112	123	64	64	64	102	70	64	105	93
S8	CA(N; 3, 5^6^)	194	NA	216	215	234	152	125	125	NA	199	NA	NA	194
S9	CA(N; 3, 6^6^)	330	343	382	373	407	300	330	331	338	336	325	350	332
S10	CA(N; 3, 5^7^)	218	229	274	236	271	201	218	218	229	236	217	233	217
S11	MCA(N; 2, 5^1^ 3^8^ 2^2^)	20	19	19	23	22	15	16	15	NA	20	20	21	20
S12	MCA(N; 2, 7^1^ 6^1^ 5^1^ 4^6^ 3^8^ 2^3^)	44	45	43	50	51	42	42	42	48	50	48	51	48
S13	MCA(N; 3, 5^2^ 4^2^ 3^2^)	114	NA	111	131	136	100*	106	108	NA	120	100	NA	113
S14	MCA(N; 3, 10^1^ 6^2^ 4^3^ 3^1^)	377	NA	383	399	414	360	361	360	385	378	382	393	355

**Table 7:** Comparison with existing strategies using CA (N; t, 2^10^), t varied from 2 to 10.

t	Computational-based Strategies							Meta-heuristic-based Strategies				
	IPOG	ITCH	Jenny	PICT`	TConfig	TVG	GTWay	PSO	HSS	HHH	CS	eFPA
2	10	6	10	NA	9	10	NA	8	7	8	8	8
3	19	18	18	NA	20	17	NA	17	16	16	16	16
4	49	58	39	NA	45	41	NA	37	37	36	36	36
5	128	NA	87	NA	95	84	NA	82	81	79	79	75
6	352	NA	169	NA	183	168	NA	158	158	153	157	157
7	NA	NA	311	NA	NA	302	NA	NA	298	NA	NA	290
8	NA	NA	521	NA	NA	514	NA	NA	498	NA	NA	495
9	NA	NA	788	NA	NA	651	NA	NA	512	NA	NA	577
10	NA	NA	1024	NA	NA	NA	NA	NA	1024	NA	NA	1024

**Table 8:** Comparison with existing strategies CA(N; 4, 5^P^), P varied from 5 to 10.

P	Computational-based Strategies									Meta-heuristic-based Strategies				
	IPOG	ITCH	Jenny	PICT	TConfig	TVG	GTWay	MIPOG	CTE-XL	PSO	HSS	HHH	CS	eFPA
5	908	837	810	773	849	731	625	779	NA	779	751	746	776	778
6	1239	1074	1072	1092	1128	1027	625	1001	NA	1001	990	967	991	985
7	1349	1248	1279	1320	1384	1216	1125	1209	NA	1209	1186	1151	1200	1166
8	1792	1424	1468	1532	1595	1443	1384	1417	NA	1417	1358	1320	1415	1319
9	1793	1578	1643	1724	1795	1579	1543	1570	NA	1570	1530	1483	1562	1465
10	1965	1791	1812	1878	1971	1714	1643	1716	NA	1716	1624	1635	1731	1592
11	2091	1839	1957	2038	2122	1852	1722	1902	NA	1902	1860	1784	2062	1719
12	2285	1964	2103	NA	2268	2022	1837	2015	NA	2015	2022	1915	2223	1854

**Table 9:** Comparison with existing strategies CA(N; 4, v^10^) with v varied from 2 to 7.

V	Computational-based Strategies									Meta-heuristic-based Strategies				
	IPOG	ITCH	Jenny	PICT	TConfig	TVG	GTWay	MIPOG	CTE-XL	PSO	HSS	HHH	CS	eFPA
2	49	58	39	43	45	40	46	43	NA	34	37	36	28	28
3	241	336	221	231	235	228	224	217	NA	213	211	207	211	208
4	707	704	703	742	718	782	621	637	NA	685	691	668	698	657
5	1965	1750	1719	1812	1878	1917	1714	1643	NA	1716	1624	1635	1731	1592
6	3935	NA	3519	3735	NA	4159	3514	3657	NA	3880	3475	3405	3894	3310
7	7061	NA	6462	NA	NA	7854	6459	5927	NA	NA	6398	6412	NA	6095

### 6.1 Evaluation of hybrid variants of FPA

In this section, the hybrid variants of FPA (i.e., original FPA, eFPA, mFPA, and lFPA) are evaluated to select the best hybrid variant algorithm. To do so, we subjected each variant to three well-known CA problems involving CA(N; 2, 10^5^), CA(N; 2, 4^6^), and CA(N; 3, 5^6^).

The results in Table 5 show that the hybrid variants of FPA outperform the original FPA in terms of average test suite size and best test suite size. The results also show that eFPA produces superior results compared with the other variants of FPA (i.e., not considering the overhead time to perform elitism). Specifically, the performance of eFPA is close to the performance of lFPA, and the performance of FPA is close to that of the mFPA. However, the results of FPA and mFPA indicate very poor performance compared with those of eFPA and lFPA.

**Fig 7:**
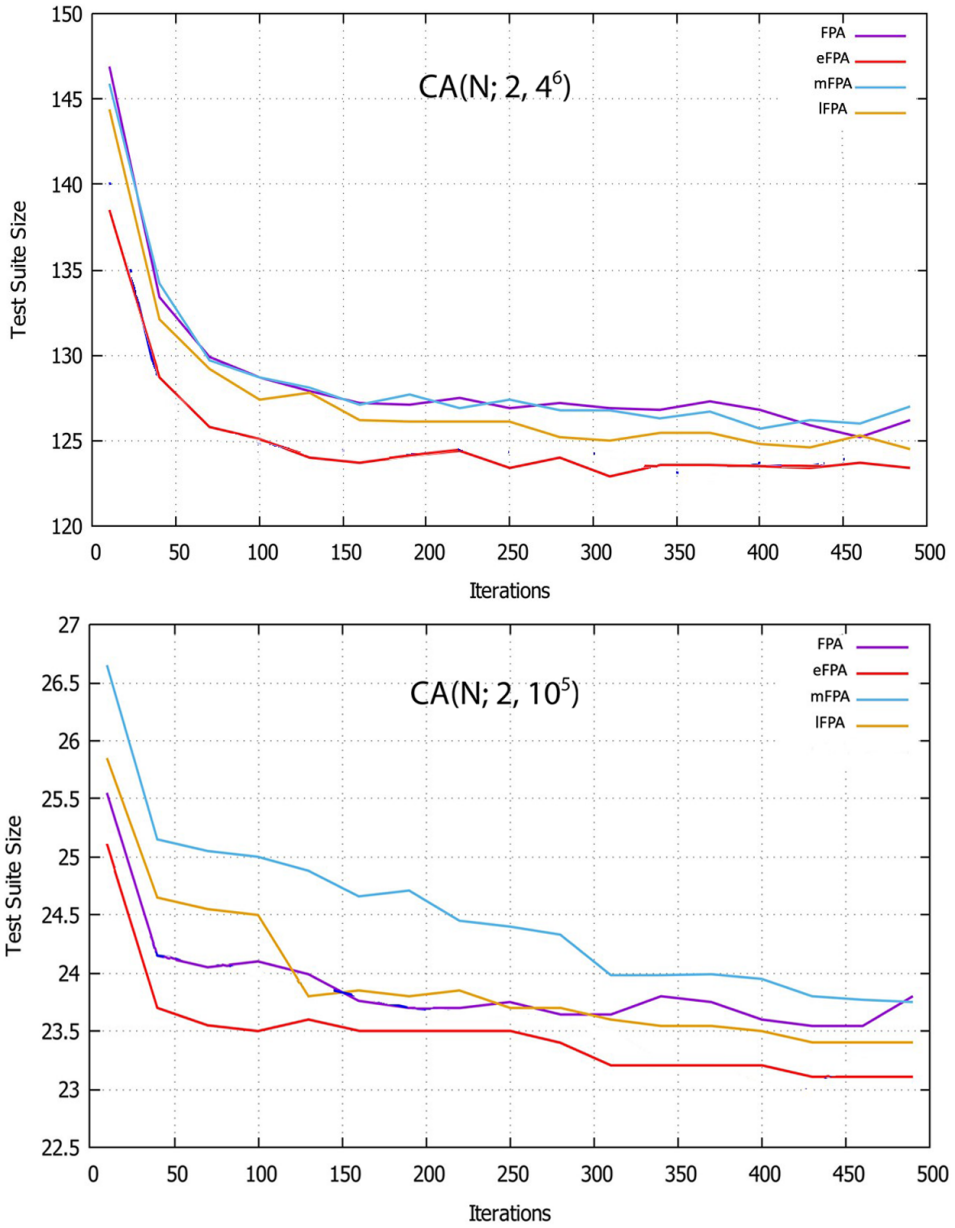
Convergence rate of hybrid variants of FPA for CA (N; 2, 10^5^) and CA(N; 2, 4^6^).

We also study the convergence rate of hybrid FPA-based strategies, which is an important aspect of any hybridization endeavor. To evaluate the convergence rate of the hybrid variants of FPA, they are executed 20 times with different iteration values (i.e., 5, 10, 20, 30, 40, 50, 100, 200, 300, 500, and 1000). The average values of the 20 runs for the two well-known CAs, CA (N; 2, 10^5^) and CA (N; 2, 4^6^), are used to demonstrate the convergence speed of the proposed algorithms. As shown in Fig 7, employing the hybridization components in the FPA improves the convergence properties. Furthermore, the convergence rates of eFPA and the combined lFPA are faster than those of the other variants.

By employing elitism, the quality of solutions in eFPA is improved. The convergence rate also improves as observed in Table 5 and Fig 7. We foresee the benefit of elitism to ensure that only the elite population is passed to the next iteration and poor solutions are replaced with random ones.

**Fig 8:**
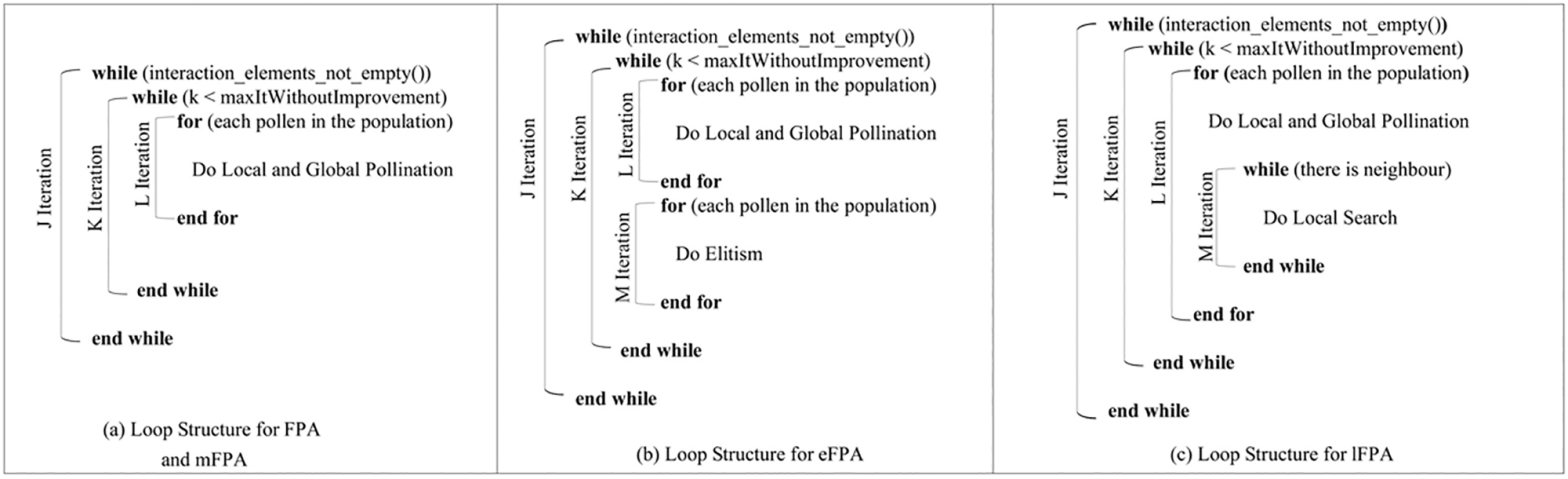
General loop structures of the original FPA, eFPA, mFPA, and lFPA.

Apart from the convergence rate, time complexity can be a useful indicator of the effectiveness of a FPA hybrid variant. Based the pseudo code excerpt in Fig 6, the loop structures for the original FPA, eFPA, mFPA, and lFPA are shown in Fig 8(a) to 8(c).

Referring to Fig 8 and assuming all other operations can be performed in a constant time, the time complexity for FPA and mFPA is *O*(*J*×*K*×*L*) ≈ *O*(*n*^*3*^) when J, K, and L are approaching a large *n*. In a similar manner, the time complexity for eFPA is *O*(*J*×*K*× (*L+M*)) ≈ O(*n*^*3*^) when J, K, L+M are approaching a large *n*. Unlike FPA, mFPA, and eFPA, the time complexity for lFPA is *O*(*J*×*K*×*L*×*M)*) ≈ O(*n*^*4*^). eFPA has better convergence while maintaining the same time complexity as the original FPA and is thus the best variant for our selection.

### 6.2 Benchmarking with existing t-way strategies

To evaluate its performance in terms of minimizing the test suite size, eFPA is compared with existing t-way strategies in terms of test suite size. Our experiment is divided into four sets of comparisons as follows:

1. Comparison of eFPA with results of strategies published in [16,17,62] for different configurations involving CA(N; 2, 3^4^), CA(N; 2, 3^13^), CA(N; 2, 10^10^), CA(N; 2, 15^10^), CA(N; 2, 5^10^), CA(N; 3, 3^6^), CA(N; 3, 4^6^), CA(N; 3, 5^6^), CA(N; 3, 6^6^), CA(N; 3, 5^7^), MCA(N; 2, 5^1^ 3^8^ 2^2^), MCA(N; 2, 7^1^ 6^1^ 5^1^ 4^6^ 3^8^ 2^3^), and MCA(N; 3, 5^2^ 4^2^ 3^2^).
2. Comparison of eFPA with existing strategies for CA (N; t, 2^10^), *t* varied from 2 to 10.
3. Comparison of eFPA with existing strategies for CA(N; 4, 5^P^), *p* varied from 5 to 10.
4. Comparison of eFPA with existing strategies for CA(N; 4, v^10^), *v* varied from 2 to 7.

Table 6 highlights the comparative results of CA(N; 2, 3^4^), CA(N; 2, 3^13^), CA(N; 2, 10^10^), CA(N; 2, 15^10^), CA(N; 2, 5^10^), CA(N; 3, 3^6^), CA(N; 3, 4^6^), CA(N; 3, 5^6^), CA(N; 3, 6^6^), CA(N; 3, 5^7^), MCA(N; 2, 5^1^ 3^8^ 2^2^), MCA(N; 2, 7^1^ 6^1^ 5^1^ 4^6^ 3^8^ 2^3^), and MCA(N; 3, 5^2^ 4^2^ 3^2^). Overall, Table 6 shows that the meta-heuristic-based strategies perform better than the computation-based strategies. Putting meta-heuristic-based strategies aside, the mAETG strategy outperforms other existing strategies in 6 out of 14 cell entries, followed by AETG, IPOG, and Jenny in 3 out of 8 cell entries, while TVG generates the worst results.

**Fig 9:**
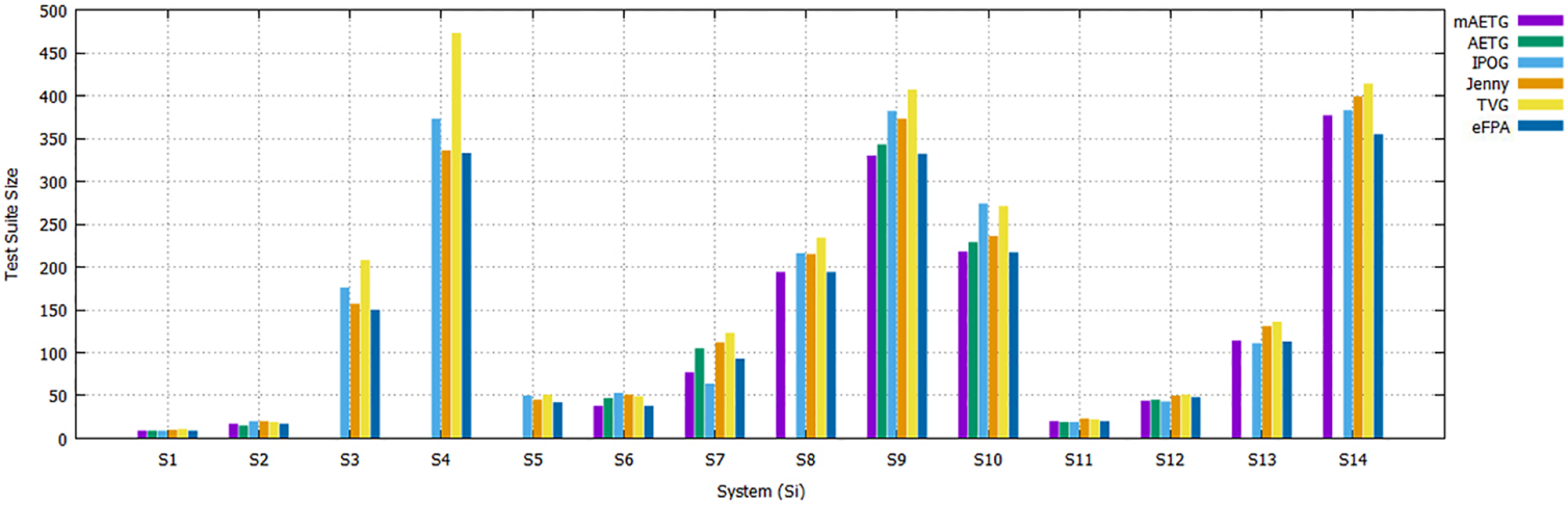
Comparison of eFPA with computational-based strategies.

**Fig 10:**
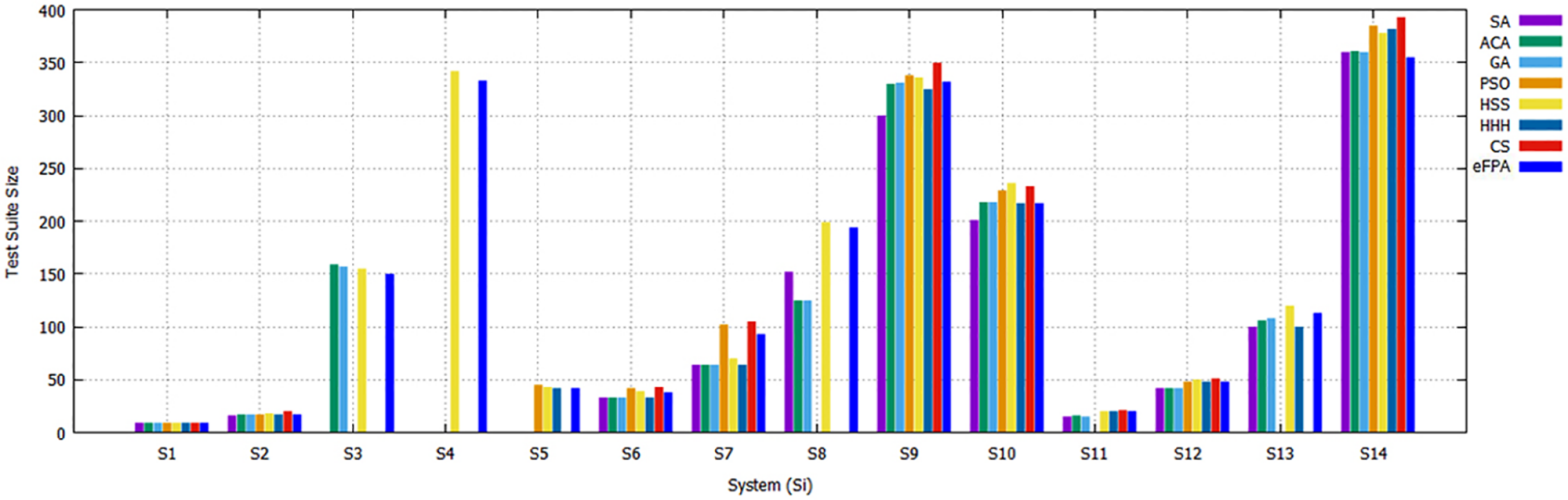
Comparison of eFPA with meta-heuristic-based strategies.

For meta-heuristic-based strategies, SA and GA outperform other existing strategies in 7 and 6 out of 14 cell entries, respectively. HHH and eFPA provide competitive performances with 5 cell entries for each, followed by ACA by 4 entries. PSO, HS, and CS perform the poorest with only 1 cell entry for PSO and HS, and no entry for CS. Thus, even though the eFPA strategy is unable to produce the smallest test suite size for all cases, Figs 9 and 10 clearly show that eFPA outperforms earlier strategies, including ACA, PSO, HS, and CS.

**Fig 11:**
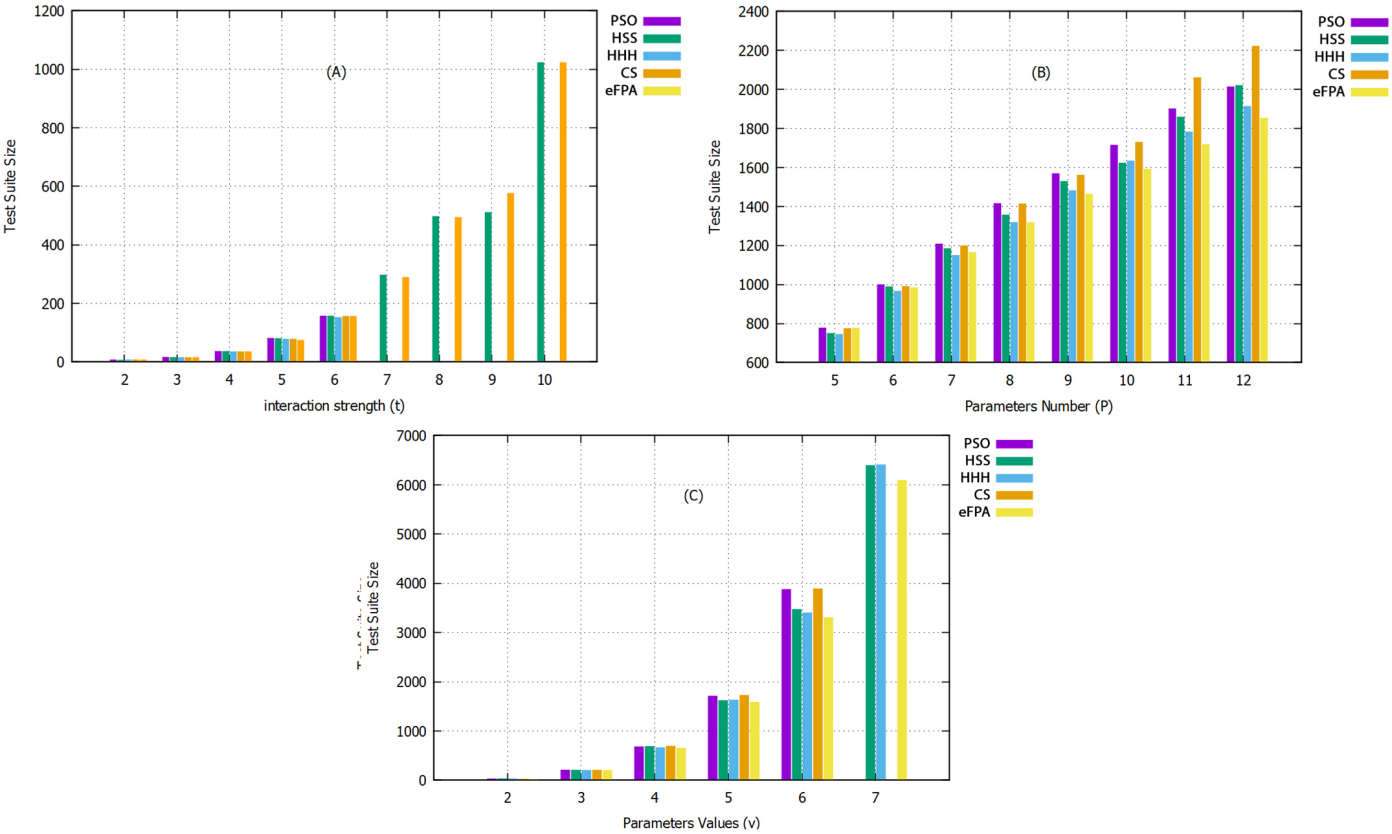
Comparison of eFPA with meta-heuristic-based strategies with: (A) *t* varied from 2 to 10, (B) *P* varied from 5 to 12, and (C) *v* varied from 2 to 7.

Table 7 highlights the case of CA (N; t, 2^10^) where t is varied from 2 to 10. Referring to Table 7, most of the existing strategies are unable to produce results beyond t > 6 due to their heavy computation (i.e., as in case of GA, ACA, GA, and PSO). eFPA and HHH have the top performance among the existing strategies (Fig 11(a)). Specifically, eFPA is ranked first by obtaining 5 out of 9 cell entries, and HHH is ranked second by obtaining 3 out of 9 cell entries. CS also provides a good performance with 2 best results out of the nine cell entries. ITCH and HS have one best entry. Meanwhile, IPOG, Jenny, PICT, TConfig, TVG, GTWay, and PSO do not have best cell entries.

Table 8 presents the results for CA(N; 4, 5^P^) where P is varied from 5 to 12. GTWay outperforms other strategies in 4 out of 8 cell entries, while eFPA outperforms other strategies in 3 entries, followed by HHH with 1 entry.

For the comparative experiment involving CA(N; 4, v^10^) with v varied from 2 to 7 in Table 9, eFPA outperforms the existing strategies in 3 out of 6 cell entries. GTWay, MIPOG, CS, and HHH come as the runner up with only one best entry. IPOG, ITCH, Jenny, PICT, TConfig, TVG, CTE-XL, PSO, and HSS perform the poorest with no best cell entry.

The results of the comparative experiments show that eFPA performs better than most existing strategies, followed by HHH, as shown in Fig 11, for the experiment results in Tables 7 to 9. Unlike eFPA, HHH offers a different kind of hybridization (i.e., hyper-heuristic approach) based on the use of four meta-heuristic algorithms. Despite having more algorithms to choose from, eFPA can still outperform HHH owing to the introduction of elitism, which lessens the effect of aggressive behavior from lévy flight motion.

### 6.3 Statistical analysis

For statistical analysis, Wilcoxon Signed Rank Test is used to analyze the significance of the results obtained. The Wilcoxon test is a non-parametric analysis technique that is used to compare two sets of ordinal data that are subjected to different conditions. In this statistic analysis, eFPA is separately compared with each existing strategy to test if a significant difference exists between the produced results of the proposed strategy and those of the other strategies. Here, we have two hypotheses:

1. Null hypothesis (*H*_*0*_), which is assumed to be true if there, is no difference between two strategies’ results.
2. Alternative hypothesis (*H*_*1*_) which is assumed to be true when there is difference between two strategies’ results, in another word when null hypothesis is false.

The experiments results show that the Wilcoxon test statistic is calculated and converted into a conditional probability called a P-value. A small P-value denotes a strong evidence to reject the null hypothesis *H*_*0*_ (i.e., no difference exists between the two strategies’ results) in favor of the alternative hypothesis. Decision-making is based on a probability threshold called Alpha (α) or significance level.

The statistics in Tables 10 and 11 provide the values of the Wilcoxon Signed Rank Test for eFPA in comparison with each strategy of our experiments. As the tables show, the Wilcoxon signed-rank test has negative ranks (i.e., number of cases that eFPA unable to outperform another strategy), positive ranks (i.e., number of cases that eFPA is better than another strategy), and ties. The column labelled Asymp. Sig. (2-tailed) shows the p-value probability; if the p-value is less than 0.005, no significant difference exists between the compared results.

**Table 10:** Wilcoxon signed rank test for experimental results from Table 6.

Pairs	Ranks				Asymp. Sig. (2-tailed)	Conclusion
	Negative Ranks	Positive Ranks	Ties	Total		
Jenny- eFPA	0	15	0	15	0.001	Reject the null hypothesis H_0_
TVG- eFPA	0	15	0	15	0.001	Reject the null hypothesis H_0_
CS- eFPA	0	8	1	9	0.012	Reject the null hypothesis H_0_
SA- eFPA	11	1	1	13	0.023	Reject the null hypothesis H_0_
PSO- eFPA	0	6	4	10	0.028	Reject the null hypothesis H_0_
mAETG- eFPA	6	2	4	12	0.035	Reject the null hypothesis H_0_
GA- eFPA	8	3	2	13	0.041	Reject the null hypothesis H_0_
IPOG- eFPA	5	8	1	14	0.087	Retain the null hypothesis H_0_
AETG- eFPA	3	5	1	9	0.092	Retain the null hypothesis H_0_
HSS- eFPA	3	10	2	15	0.141	Retain the null hypothesis H_0_
ACA- eFPA	8	3	2	13	0.168	Retain the null hypothesis H_0_
HHH- eFPA	4	1	6	11	0.345	Retain the null hypothesis H_0_

**Table 11:** Wilcoxon signed rank test for experiments results from Tables 7 till 9.

Pairs	Ranks				Asymp. Sig. (2-tailed)	Conclusion
	Negative Ranks	Positive Ranks	Ties	Total		
Jenny—eFPA	0	22	1	23	0.000	Reject the null hypothesis H_0_
GTWay—eFPA	12	2	0	14	0.002	Reject the null hypothesis H_0_
MIPOG—eFPA	12	2	0	14	0.002	Reject the null hypothesis H_0_
HSS—eFPA	4	17	2	23	0.007	Reject the null hypothesis H_0_
PICT`—eFPA	9	4	0	13	0.013	Reject the null hypothesis H_0_
CS—eFPA	11	3	4	18	0.013	Reject the null hypothesis H_0_
ITCH—eFPA	10	5	0	15	0.021	Reject the null hypothesis H_0_
PSO—eFPA	10	7	1	18	0.029	Reject the null hypothesis H_0_
IPOG—eFPA	10	9	0	19	0.044	Reject the null hypothesis H_0_
HHH—eFPA	11	5	3	19	0.052	Retain the null hypothesis H_0_
TVG—eFPA	10	12	0	22	0.115	Retain the null hypothesis H_0_
TConfig—eFPA	8	8	0	16	0.179	Retain the null hypothesis H_0_

Table 10 depicts the Wilcoxon signed-rank test for the experimental results in Table 6. The results are statistically significant in Jenny, TVG, CS, SA, PSO, mAETG, and GA but not in AETG, IPOG, ACA, HSS, and HHH. Despite showing statistical significance in only half of the cases, the positive ranks of eFPA are higher than its negative ranks.

The statistical analysis of the experiment results in Tables 7 to 9 is depicted in Table 10. The null hypothesis, H_0_, is rejected in most of cases. The finding proves that eFPA has a statistically better test suite size than the other strategies.

## 7. Threats to validity

Most experimental studies encounter threats to validity. In our case, the fairness of the benchmark experiments can be an issue owing to the unavailability of source codes and their corresponding implementation. As such, the time performance cannot be fairly compared between strategies as the running environments, the data structure, the implementation language, and the operating environments are different. Thus, the time performances have been dropped.

Another threat to validity relates with the meta-heuristic-based strategies. Maximum iteration and population size typically affect the test size performance, that is, the probability of getting better results typically increases with the iteration and population size. In our experiments, we assume that the existing meta-heuristic-based strategies have been sufficiently tuned to obtain the best possible results (regardless of their maximum iteration and population size).

Finally, meta-heuristic-algorithms often rely on randomization to generate the population update. As such, the reported best results may be obtained by chance and may affect our conclusion.

## 8. Conclusion and further work

In this paper, we propose a new t-way test suite strategy based on the FPA. Then, we propose three hybridizations variants for the FPA. The hybridization variants are obtained by grafting the elitism, mutation operator, and local search components into the FPA strategy. Experiment results show that the elitism-FPA-based strategy (eFPA) performs better than the other variants. The eFPA is compared with existing strategies in the context of t-way test suite generation. In many cases, the eFPA outperforms the other strategies. In the case where eFPA fails to produce optimum results, the results are still within reasonable values. Owing to the encouraging results, we are looking to adopt the eFPA for variable strength t-way testing and explore the possibilities of constraints-based software product lines.
